# Supplementary material for: Centromeric KIR AA Individuals Harbor Particular KIR Alleles Conferring Beneficial NK Cell Features with Implications in Haplo-Identical Hematopoietic Stem Cell Transplantation
Source: Cancers (Basel). 2020 Dec 1;12(12):3595. doi: 10.3390/cancers12123595 (PMC7760878; doi:10.3390/cancers12123595)
Supplement: Supplementary file 1 [file cancers-12-03595-s001.pdf]

a

Domains	D1						D2													
Residue	16	21	44	45	68	72	105	106	114	132	133	134	135	154	163	181	182	183	184	187
Consensus	R	E	M	F	R	D	Y	E	P	S	S	Y	D	P	D	F	H	D	S	E
KIR2DL1*002	P	-	-	-	-	-	-	-	-	-	-	-	-	-	-	-	-	-	-	-
KIR2DL1*003	-	-	-	-	-	-	-	-	L	-	-	-	-	-	-	-	-	-	-	-
KIR2DL1*004	-	-	-	-	-	-	-	-	-	-	-	-	-	T	N	-	R	-	-	-

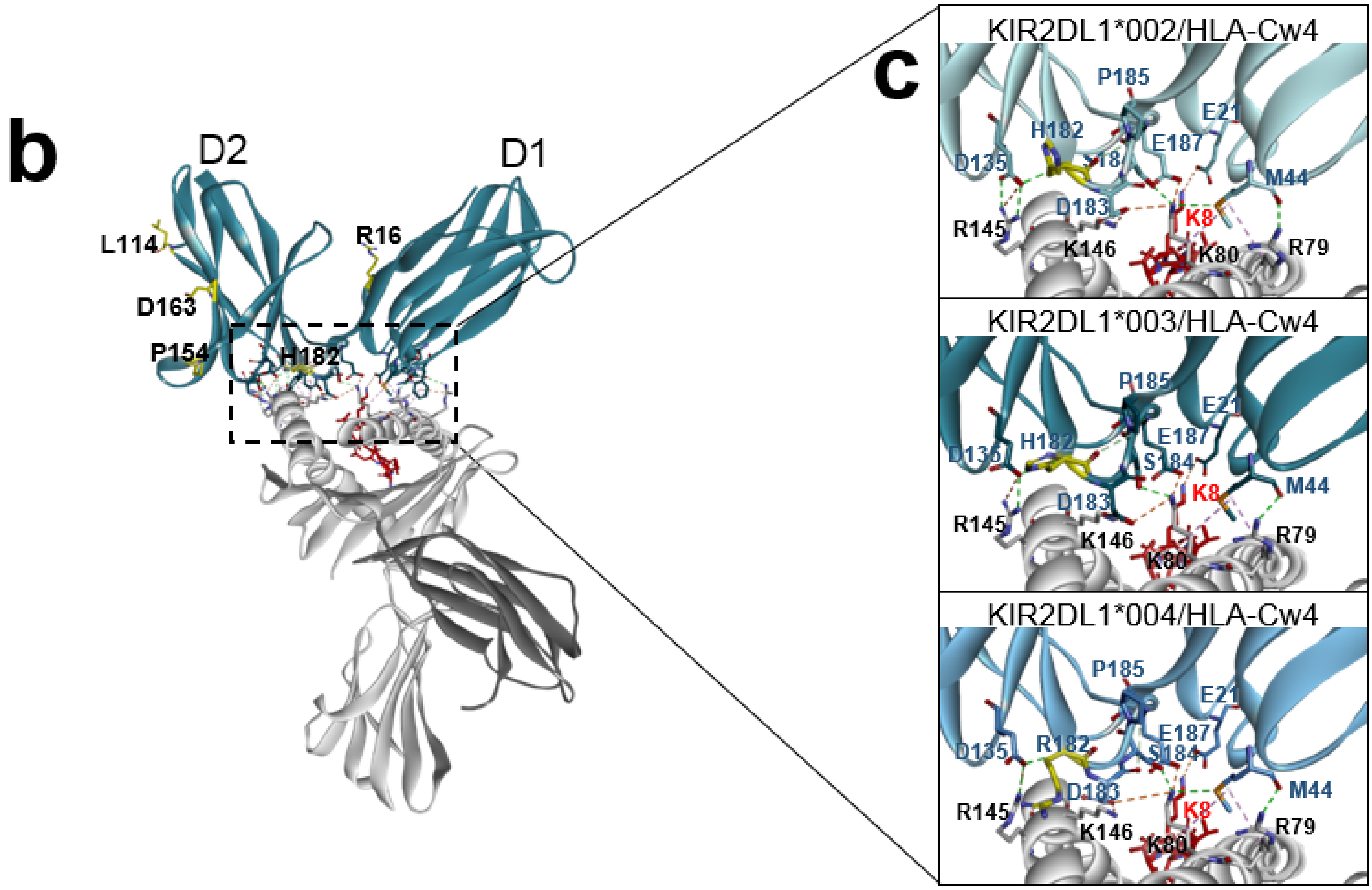

Figure S1. Structural model of KIR2DL1 allotypes complexed with HLA-Cw4 molecule. (a) Alignment of partial amino acid sequences of D1 and D2 domains for three KIR2DL1 allele-encoded receptors (allotypes). Identity with the consensus sequence is indicated by dashes "– ", the polymorphic residues are highlighted in grey and residues in contact with the HLA-Cw4 molecule are also shown. **(b)** Ribbon diagram of the KIR2DL1*003 allotype (in blue) bound to the HLA-Cw4 molecule (in grey) and in complex with its specific peptide (in red) (modelling based on the structure PDB 1IM9). The polymorphic residues are shown in yellow. **(c)** Details and comparison of the interaction network between three KIR2DL1 allotypes (in blue) and the HLA-Cw4 molecule (in grey) in complex with its specific peptide (in red), the polymorphic residue at position 182 of the KIR2DL1 allotypes is shown in yellow.

Table S1. Numerous KIR2DL1/2/3/S1/S2 allele combinations encountered in a cohort of French blood donors (N=108) depending on KIR gene motifs and HLA-Cw environment

	KIR allele typing											HLA-C environment			KIR motifs	
	2DS2	2DS2	2DL2	2DL2	2DL3	2DL3	2DL1	2DL1	2DS1	2DS1	N	C1C1	C1C2	C2C2	Cen	Tel
1	-		-		__*001__		__*003__		-		9	4	4	1	AA	AA
2	*001		___*001___		__*001__		__*003__	___*004___	*002		6	1	3	2	AB	AB/BB
3	-		-		__*002__	*007	__*002__		-		5	4	1		AA	AA
4	*001		*003		__*002__		__*002__		-		5	3		2	AB	AA
5	*001		___*001___		__*002__		__*002__	___*004___	*002		5	1	4		AB	AB/BB
6	-		-		+		*003		-		4	1	3		AA	AA
7	-		-		__*002__	___*001___	__*002__	___*003___	-		4	2		2	AA	AA
8	*001		___*001___		__*002__		__*002__	___*004___	-		4	4			AB	AA/AB
9	*001		*001		__*002__		__*002__		-		4	2	1	1	AB	AA/AB
10	*001		*003		__*001__		__*003__		-		3	2		1	AB	AA
11	*001		___*001___		__*001__		__*003__	___*004___	-		3		1	2	AB	AA/AB
12	*001		*003		__*002__		__*002__		*002		3	2	1		AB	AB/BB
13	-		-		__*002__	___*005___	__*002__	___*001___	-		2		1	1	AA	AA
14	-		-		*005	*007	*002		-		2		1	1	AA	AA
15	-		-		__*001__	*002	__*003__		-		2	1	1		AA	AA
16	*001		*001		__*001__		__*003__		*002		2		1	1	AB	AB/BB
17	*001		+		+		*003	*004	*002		2		1	1	AB	AB/BB
18	*001		__*003__	*001	-		__*004__		-		2	1		1	BB	AA
19	-		-		__*001__	___*005___	__*003__	___*001___	-		1	1			AA	AA
20	-		-		*002	*015	*003	*001	-		1		1		AA	AA
21	-		-		__*002__		__*002__		-		1	1			AA	AA
22	-		-		__*002__	*001	__*002__	001/002/00	-		1		1		AA	AA
23	-		-		__*001__		__*003__	*001/002	-		1	1			AA	AA
24	-		-		+		*003	*001/002	-		1		1		AA	AA
25	-		-		__*001__	*002	__*003__	001/002/00	-		1		1		AA	AA
26	-		-		__*002__		__*002__		*002		1		1		AA	AB
27	-		-		__*002__	*007	__*002__		*002	*003	1	1			AA	AB
28	-		-		__*002__	*001	__*002__	*004	*002		1			1	AA	AB
29	-		-		__*001__		__*003__		*002	*006	1	1			AA	AB
30	-		-		__*001__		__*003__		*002	*003	1		1		AA	AB
31	-		-		*003		*003		*002		1			1	AA	AB
32	-		-		__*001__		__*003__		*002		1	1			AA	AB
33	-		-		+		*003	001/002/00	*002		1		1		AA	AB
34	-		-		__*001__	*005	__*003__	*001/002	*002		1		1		AA	AB
35	-		-		__*001__	*002	__*003__		*002	*006	1	1			AA	AB
36	-		-		__*001__	*002	__*003__		*002		1			1	AA	BB
37	-		-		+		*003	*004	-		1		1		AA	nd
38	*001		__*001__		___*005___		__*004__	___*001___	-		1			1	AB	AA
39	*001		*001		__*002__		__*002__	*007	-		1		1		AB	AA
40	*001		+		+		*002	*007	-		1			1	AB	AA
41	*001		*012		__*001__		__*003__		-		1	1			AB	AA
42	*001		*001		__*001__		__*003__	*004/006	-		1	1			AB	AA
43	*001		*003		__*002__		__*002__		*002	*003	1		1		AB	AB
44	*001		__*001__		+		*002	__*004__	*002		1	1			AB	AB
45	*001		*008		__*002__		__*002__	*004	*002		1			1	AB	AB
46	-		+		__*001__		__*003__		*002		1	1			AB	AB
47	*001	*002	___*001___		__*001__		__*003__	___*004___	*002		1		1		AB	AB
48	*001		+		+		*003	*004	-		1		1		AB	AB
49	*001		+		+		*003	*007	*002		1	1			AB	BB
50	*001		*003		-		-		-		1		1		BB	AA
51	*001		*003	*012	-		-		-		1		1		BB	AA
52	*001		*003	*012	-		-		*002/006		1	1			BB	AB
53	*001	*002	__*003__	*008	-		__*004__		*002		1	1			BB	AB
54	*001		*001		-		*004		-		1			1	BB	AB
55	*001		*001	*008	-		*004		-		1			1	BB	AB
56	*001		*001	*005	-		*004		-		1		1		BB	AB
57	*001		__*003__		-		__*004__		*002		1		1		BB	AB
58	*001		+		-		*007	*001/002	*002		1		1		BB	AB
59	*001		+		-		*007		*002		1			1	BB	AB
_N_											_108_	_42_	_41_	_25_		

KIR2DL1/2/3/S1/S2 alleles were assigned using NGS and Profiler software. Main KIR2DL1/2/3 allele combinations were highlighted in bold or italics characters. Absence of a specific KIR gene was mentioned using "-". KIR2DL1/2/3 remaining allele ambiguities and non-assigned alleles were indicated using "/" and "+" respectively. KIR centromeric (Cen)/telomeric (Tel) motifs were defined. C1C1, C1C2 and C2C2 environment were defined depending on HLA-C allelic typing.

Table S2. Characteristics of patients

	All patients N=81	CenAA* donors N=39	CenAB.BB* donors N=42	*P* value
Gender: male	49 (60%)	26 (67%)	23 (55%)	0.36
Median age: years (range)	60 (24-71)	61 (24-71)	58 (32-71)	0.71
Disease				
AML/MDS	32/14 (57%)	17/6 (59%)	15/8 (55%)	0.82
ALL	5	1	4	
HD	4	2	2	
NHL	14	7	7	
CLL	4	3	1	
Myelofibrosis	4		4	
pDCs neoplasm	2	2		
CML	1		1	
Mycosis fungoides	1	1		
Myeloid/lymphoid	51(63%)/30(37%)	23(59%)/16(41%)	28(67%)/14(33%)	0.50
Status at treatment				
CR1/CR2/CR3	30/10/3 (53%)	13/4/1 (46%)	17/6/2 (60%)	0.27
PR1/PR2/PR3/PR5	5/5/4/4 (22%)	3/3/3/4 (33%)	2/2/1 (12%)	
Active	19 (24%)	7 (18%)	12 (28%)	
Aplasia	1 (1%)	1 (3%)		
Disease risk index				
Intermediate	34 (42%)	15 (38%)	19 (45%)	0.65
High	47 (58%)	24 (62%)	23 (55%)	
Previous allograft	12 (15%)	6 (15%)	6 (15%)	
Conditioning				
Baltimore	26 (32%)	11 (28%)	15 (36%)	0.49
Clo-Baltimore	27 (33%)	13 (33%)	14 (33%)	
CloB2A1	28 (35%)	15 (38%)	13 (31%)	
Haplo-donors				
Median age: years (range)	42 (20-72)	42 (22-71)	41 (20-72)	0.85
Sister/Brother	12/20 (40%)	9/7 (41%)	3/13 (38%)	0.83
Father/Mother	6/2 (10%)	4/1 (13%)	2/1 (7%)	
Son/Daughter	24/12 (44%)	11/4 (38%)	13/8 (50%)	
Nephew	5 (6%)	3 (8%)	2 (5%)	
Donor/recipient CMV status				
-/-	46 (57%)	20 (51%)	26 (62%)	0.37
-/+	16 (20%)	8 (21%)	8 (19%)	
+/-	8 (10%)	5 (13%)	3 (7%)	
+/+	11 (13%)	6 (15%)	5 (12%)	
ABO compatibility				
compatibility	53 (65%)	27 (70%)	26 (62%)	0.64
Minor inc	15 (19%)	6 (15%)	9 (21%)	
Major inc	13 (16%)	6 (15%)	7 (17%)	
Graft composition				
Median $CD34^+$ cells: 10*6/kg	7.92 (2.88-19.94)	7.89 (3.10-15.16)	7.96 (2.88-19.94)	0.95
Median $CD3^+$ T cells: 10*7/kg	26.61 (7.71-49.99)	25.02 (12.60-46.66)	24.27 (7.71-49.99)	0.94
Median $CD45^+$ cells: 10*8/kg	8.57 (3.80-18.65)	8.04 (3.80-18.65)	9.07 (5.04-13.80)	0.80

Abbreviations: AML: acute myeloid leukemia; MDS: myelodysplastic syndrome; ALL: acute lymphoblastic leukemia; HD: Hodgkin disease; NHL: non-Hodgkin lymphoma; CLL: chronic lymphocytic leukemia; pDCs: plasmacytoid dendritic cells; CML: chronic myeloid leukemia; CR: complete remission; PR: partial remission; CMV: cytomegalovirus; inc: incompatibility. *CenAA donors are KIR2DL3+/2DL2-/S2- ; CenAB.BB donors are KIR2DL2/S2+ and KIR2DL3-/+
